# Genetic Risk Assessment of Degenerative Eye Disease (GRADE): study protocol of a prospective assessment of polygenic risk scores to predict diagnosis of glaucoma and age-related macular degeneration

**DOI:** 10.1186/s12886-023-03143-5

**Published:** 2023-10-24

**Authors:** Georgina L Hollitt, Ayub Qassim, Daniel Thomson, Joshua M Schmidt, Thi Thi Nguyen, John Landers, Stuart MacGregor, Owen M Siggs, Emmanuelle Souzeau, Jamie E Craig

**Affiliations:** 1 Department of Ophthalmology, Flinders University, 1 Flinders Drive, 5042 Bedford Park, SA Australia; 2 QIMR Berghofer Medical Research Institute, 4006 Herston, QLD Australia; 3 Garvan Institute of Medical Research, 2010 Darlinghurst, NSW Australia

**Keywords:** Glaucoma, Polygenic risk score, Genetic testing, Macular degeneration, POAG, AMD

## Abstract

**Background:**

Glaucoma and age-related macular degeneration (AMD) account for a substantial portion of global blindness. Both conditions are highly heritable, with recognised monogenic and polygenic inheritance patterns. Current screening guidelines lack decisive recommendations. Polygenic risk scores (PRS) allow for cost-effective broad population risk stratification for these conditions. The predictive potential of PRS could facilitate earlier diagnosis and treatment, and prevent unnecessary vision loss.

**Methods:**

The Genetic Risk Assessment of Degenerative Eye disease (GRADE) study is a prospective study designed to generate high-quality evidence about the feasibility of PRS to stratify individuals from the general population, enabling identification of those at highest risk of developing glaucoma or AMD. The targeted recruitment is 1000 individuals aged over 50 years, from which blood or saliva samples will be used for genotyping and an individual PRS for glaucoma and AMD will be derived. Individuals with PRS values in the bottom decile (n = 100), top decile (n = 100) and middle 80% (n = 100) for both glaucoma and AMD will undergo a detailed eye examination for glaucoma and/or AMD.

**Discussion:**

The primary objective will be to compare the prevalence of glaucoma and AMD cases between low, intermediate, and high PRS risk groups. We expect to find a higher prevalence of both diseases in the high PRS risk group, as compared to the middle and low risk groups. This prospective study will assess the clinical validity of a PRS for glaucoma and AMD in the general Australian population. Positive findings will support the implementation of PRS into clinical practice.

## Background

Glaucoma and age-related macular degeneration (AMD) are the two most common causes of irreversible vision loss among elderly people worldwide [1, 2]. With the ageing population, these diseases will pose an increasingly significant burden. Furthermore, sight is generally considered to be the most valued sense by the general public, so identifying cost-effective screening methods to facilitate early diagnosis, prevention, and timely intervention is important [3]. In Australia, vision impairment results in significant direct and indirect health care costs, ranking as the seventh most costly health condition [4]. It is important to also consider the impact of vision loss on an individual, which can result in poorer wellbeing outcomes through the impact on quality of life, lost income, and personal healthcare costs [4].

Glaucoma is predicted to affect up to 111.8 million people worldwide by 2040 [2]. The condition results in irreversible vision loss due to progressive optic nerve damage. Primary open angle glaucoma (POAG) is the most common form of the disease, characterised by a normal, open anterior chamber drainage angle [5]. Accepted risk factors for POAG are both genetic and non-genetic. Risk factors with a genetic basis include increasing age, African ancestry, a positive family history, and elevated intraocular pressure (IOP) [6, 7]. The only known modifiable risk factor is raised IOP which is often, but not always, associated with the development of POAG, and IOP-lowering treatment modalities are effective at preventing or slowing disease progression [8]. Glaucoma is usually asymptomatic in the early stages, although progressive vision loss can lead to blindness if left untreated. Current screening methods are inadequate as approximately half of those with glaucoma are undiagnosed [9]. Glaucoma is one of the most heritable common complex conditions, with heritability estimated at 80% [10]. Both highly penetrant rare variants and common variants with much smaller effect sizes, have been associated with POAG [10]. Rare variants in genes including *MYOC*, *TBK1* and *OPTN*, account for less than 5% of POAG cases, [11] with common variants therefore thought to explain the majority of POAG genetic risk.

Similar to glaucoma, AMD is a common eye condition, with a reported prevalence of 13% in those aged over 85 years [1] and is predicted to affect 288 million people by 2040 [12]. It is a progressive condition that causes degeneration of the macula, leading to central vision loss. AMD is asymptomatic in its early stages, with variable progression to visually significant advanced disease depending on clinical and environmental factors [13]. Recognised risk factors for AMD include increasing age, smoking and genetic predisposition [1]. Advanced AMD is classified as either non-neovascular (dry AMD) or neovascular (wet AMD) based on the presence or absence of choroidal neovascularisation. Currently, dry AMD management relies on lifestyle modifications such as smoking cessation and dietary supplementation, [14] while wet AMD is treated with intravitreal injections of vascular endothelial growth factor (VEGF) inhibitors, a key modulator of neovascularisation [15]. Importantly, treatment with VEGF inhibition must be implemented in a timely fashion from the onset of exudative disease. Although some environmental risk factors are well recognised, research indicates there is a strong genetic basis for AMD [1]. Genetic factors may explain variance in disease severity, with heritability estimated at 45–70% [17].

Screening for glaucoma and AMD is largely opportunistic, and broad community screening has not been demonstrated to be cost-effective [16, 17]. For this reason, identifying cost-effective screening methods to facilitate early diagnosis and timely intervention is important. The National Health and Medical Research Council (NHMRC) in Australia currently recommends screening with a clinical examination for first-degree relatives of patients with glaucoma, commencing 5–10 years earlier than the age of glaucoma onset in their affected relative. Additionally, screening from the age of 40 years is recommended in people of African ancestry, compared to from 50 years of age in people of European ancestry [16]. There are no similar recommendations for AMD.

Polygenic risk scores (PRS) are an emerging clinical tool which offer a unique opportunity to improve disease risk prediction for complex heterogeneous diseases, such as glaucoma and AMD [18]. Genome-wide association studies (GWAS) have led to the identification of genetic variants, in the form of single nucleotide polymorphisms (SNPs), which are associated with a disease phenotype. Each SNP confers a different effect on disease risk, with the effect size of each SNP derived from its strength of association with a disease or disease trait in large cohort studies. A PRS summarises this genetic information into an accessible tool to quantify the genetic risk for complex genetic diseases. A PRS is the sum of independent risk alleles an individual carries, weighted by the effect size of each variant [19]. The normal distribution of a PRS, allows risk to be classified into equal groups of frequency distribution [19]. Clinically, quantiles allow for easy assessment of where an individual lies on the population distribution. Ultimately, this score can be used in addition to conventional risk factors to estimate overall disease risk rather than diagnose diseases.

Large GWAS have identified a significant number of common genetic variants associated with POAG or its endophenotypes [20]–[24]. The collective impact of these common variants on glaucoma risk, in the form of a glaucoma PRS, has been effective in stratifying risk within the general population, as well as predicting structural progression and the likelihood of requiring surgical intervention in those with already diagnosed glaucoma [20]. In the subset of patients with monogenic variants associated with glaucoma (*MYOC* variant (p.Gln368Ter), the PRS can further stratify individuals into high versus low risk groups [20, 10]. Common and rare variants have also been implicated in AMD risk through GWAS [25, 26]. An AMD PRS using 52 variants showed a 44-fold increased risk of developing AMD for those in the top decile compared to the bottom decile [25]. Furthermore, this PRS was associated with more rapid disease progression [27, 28]. The discovery of genetic associations has also helped to reveal underlying pathophysiologic mechanisms of AMD, exposing potential new treatment targets [29].

With the ability to identify those at highest risk of disease, as well as estimating disease severity and treatment response, there is potential to offer personalised care for glaucoma and AMD patients. This predictive approach could facilitate an exciting change in disease screening and treatment, and ultimately lead to a reduction in vision loss caused by these common conditions. Here we present a prospective population-based study which will assess the prevalence of both glaucoma and AMD across their relative PRS spectra. This will be the first study to assess the clinical validity of a PRS for glaucoma and AMD for clinical implementation in a real-world setting.

**Fig. 1:**
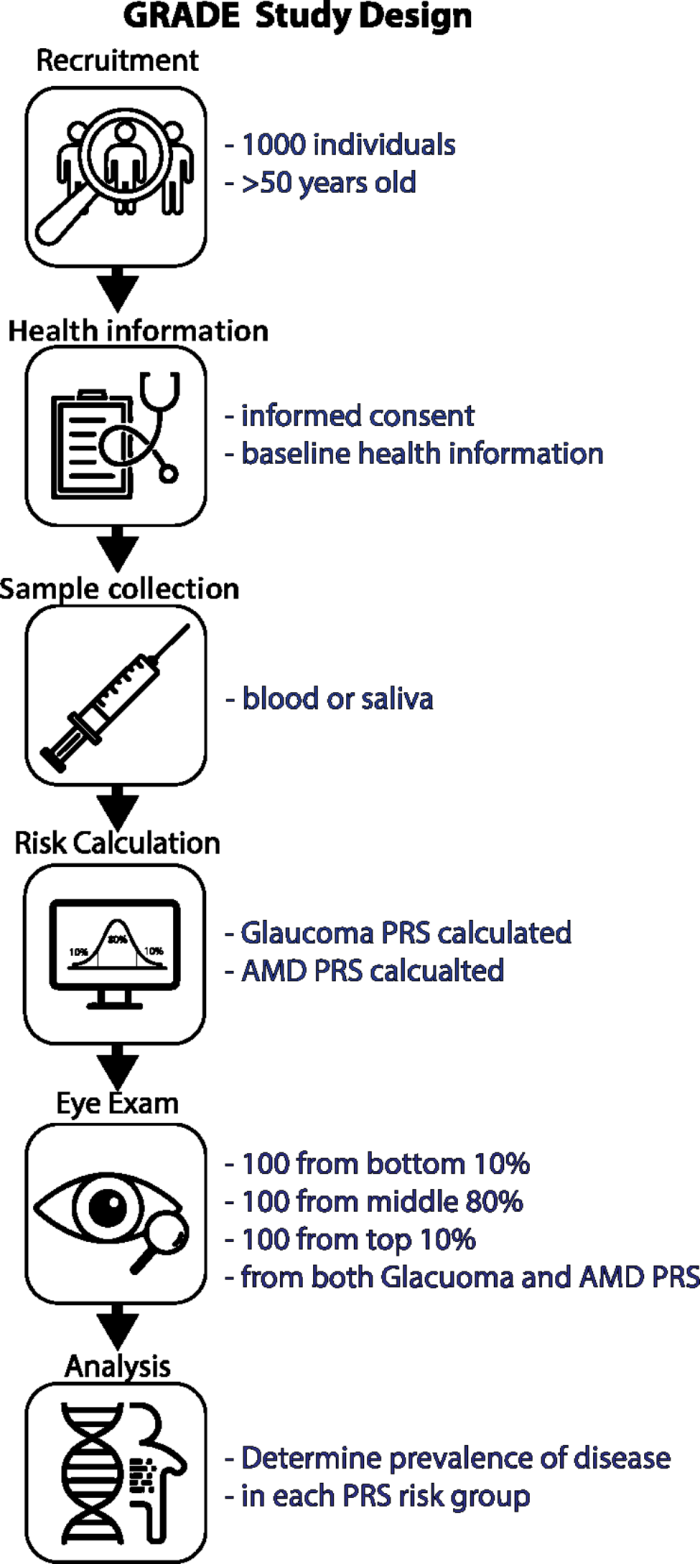
Study design

**Fig. 2:**
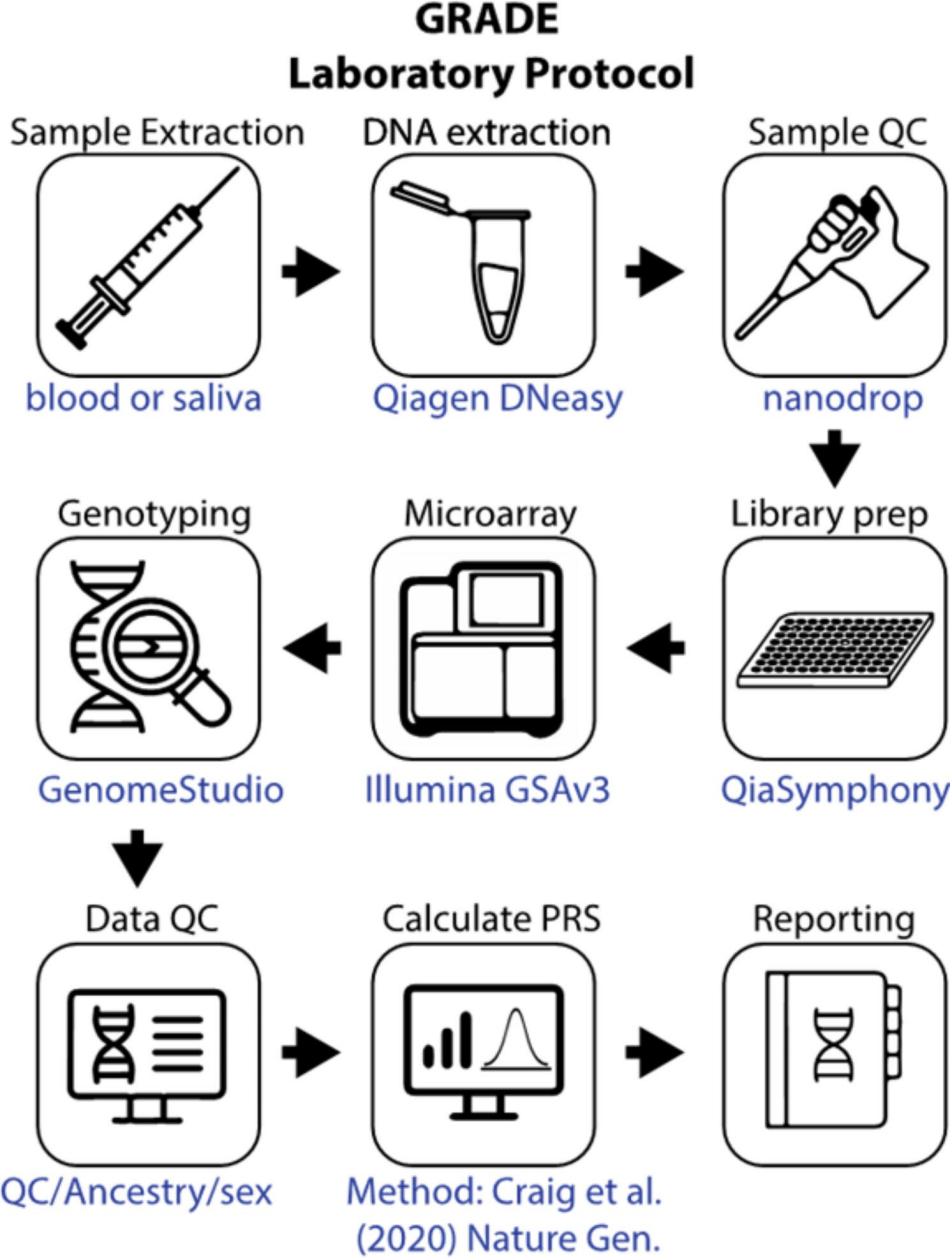
Flowchart of PRS calculation framework

## Study design

This prospective cohort study was approved by the Southern Adelaide Clinical Human Research Ethics Committee (SAC HREC) and adheres to the Revised Declaration of Helsinki. The study design is summarised in Fig. 1. The research is being conducted at the Department of Ophthalmology at Flinders University, the QIMR Berghofer Medical Research Institute and Seonix Bio under separate ethics approvals and agreements.

## Methods

### Study objectives and hypotheses

The study will apply PRS testing in 1000 individuals over the age of 50 years from the general population, and then examine a subset of individuals across the PRS spectrum with the aim of ascertaining all cases of glaucoma and AMD. We will prospectively assess the clinical validity of a PRS in stratifying high and low risk individuals, and hypothesise that there will be a higher prevalence of glaucoma and AMD in the high risk PRS groups compared to the middle and low risk groups.

### Participants

Participant recruitment methods are compliant with the Health Care Act 2008. A minimum of 1000 individuals over the age of 50 years will be invited to participate. Glaucoma and AMD prevalence increases with age, with prevalence rates commonly reported from 50 years of age [1, 2, 12]. Consequently, identifying early or established disease in individuals across the risk spectrum will be easier for individuals within this age range. Exclusion criteria include age under 50 years, or an inability to provide written informed consent. Individuals already diagnosed with glaucoma and/or AMD will not be excluded, nor will they be targeted. Recruitment will be unselected to include individuals of any ethnicity.

Potential participants will be identified using several approaches. All eligible individuals who participated in a questionnaire-based study of individuals without glaucoma assessing attitudes towards polygenic risk testing for glaucoma will be invited to participate in this study [30]. A flyer advertising the project will be displayed in public and private outpatient clinics, and sporting venues and community clubs, provided to social/community organisations and distributed via email to these groups. Presentations about degenerative eye disease will be given to community organisations to promote interest and stimulate recruitment from the general population. Individuals in outpatient clinics will be approached in person and invited to participate if the inclusion criteria are met. Demographic and health information recorded for each participant will include past medical, ocular and medication history. Individuals with a personal or family history of glaucoma or AMD may be more likely to respond to advertisements, however selection bias will largely be mitigated by wide and non-selective recruitment from all other avenues.

### Participation requirements

Participation requires individuals to provide a blood sample (2 × 9ml EDTA tubes) or a saliva sample (Oragene OG-500 collection tube, DNA Genotek, Ottawa, Ontario, Canada). A subset of participants will be invited to undergo a detailed eye examination for glaucoma and/or AMD. Eye examinations will be performed on 100 individuals each in the bottom 10%, top 10%, and middle 80% of the PRS distributions for glaucoma and AMD. Individuals undergoing eye examinations will be randomly selected within their respective PRS grouping. In total 300 participants will be examined for each disease, with a maximum of 600 participants being examined. In practice, some participants will be selected to be examined for both their glaucoma and AMD PRS results, so the number of participants undergoing eye examinations will be less than 600.

### Genetic studies

The laboratory protocol is summarised in Fig. 2. Genomic DNA will be extracted using column-based DNA purification protocols (Qiagen DNeasy) from either blood or saliva samples. Both blood and saliva will be considered viable alternatives for DNA extraction. De-identified samples of extracted DNA will be provided to a genotyping provider for array-based genotyping. Samples will be genotyped on Illumina GSA v3 arrays, with genotype imputation performed locally with Minimac3 using the 1000 Genomes data as a reference panel. Imputation and derivation of glaucoma PRS values will be performed in the laboratory of S.M. using the multitrait analysis of GWAS (MTAG) glaucoma PRS described in detail elsewhere, [20] and the pipelines developed by Seonix Bio. All individuals will have their PRS percentile determined from the relevant 1000 Genomes population, [31] with individual ancestry based on estimates from principal components derived from the genome-wide genetic data. Depending on the distribution of ancestries within the cohort, a sub-analysis may then be performed comparing outcomes between European and non-European groups. Imputation and derivation of AMD PRS values will also be performed by S.M. using a MTAG AMD PRS described in detail elsewhere [25, 26].

### Eye examinations

Clinical eye examinations will be performed on 100 individuals from each of the bottom decile, top decile, and the middle 80% of the PRS distributions for glaucoma and AMD. Individuals will be selected using random sampling methods. Examinations will include best-corrected visual acuity, IOP (as measured by Goldmann applanation tonometry), corneal pachymetry, 24 − 2 Humphrey automated perimetry, spectral domain optical coherence tomography (OCT) of the optic disc and macula, fundus autofluorescence, anterior segment OCT, stereo-disc and fundus photography [32]. All clinical investigation results will be reviewed by independent clinicians who will determine their glaucoma or AMD classification by consensus. Examiners and clinicians reviewing results will be blinded to individuals’ PRS results. Glaucoma diagnostic classification will follow previous definitions used in the PROGRESSA study [33]. Each eye will be classified as either normal examination, glaucoma suspect, open-angle glaucoma (OAG) or non-OAG (e.g. primary closed angle glaucoma). For AMD, each eye will be classified as either no AMD or normal ageing changes, early AMD, intermediate AMD, or late AMD.

### Sample size and power calculations

Using data from the UK Biobank (age at ICD-10 or self-reported glaucoma diagnosis), we estimate that ~ 3% of individuals will have a glaucoma diagnosis by the age of 64 years (Fig. 3D in reference [20]). Assuming an equal representation of subjects across all age groups, and assuming that 50% of glaucoma is undiagnosed in the community, [9] we expect ~ 10% of individuals in the top decile will have glaucoma, compared to ~ 3% in the bottom decile. The proportion of glaucoma suspects is expected to be more than 2 times the glaucoma cases based on the same preliminary analyses [9]. Based on the combined estimated incidence of glaucoma plus glaucoma suspect cases in each group (i.e. 30% in the top decile vs. 9% in the bottom decile), the current sample size will yield > 95% power (⍺=0.05) to detect a significant difference between the top and bottom deciles of the PRS distribution (logistic regression of glaucoma status on PRS decile).

Similar analyses for AMD suggest a disease prevalence of 0.7% in the bottom decile, and 22.7% in the top decile, [25] within a general population above 75 years of age and a disease prevalence of 5%. Australian epidemiological studies have estimated an AMD population prevalence of 14.3% in individuals aged 49 years and over, [34] so we expect to be sufficiently powered to detect a significant difference between the top and bottom PRS deciles at > 80% power (⍺=0.05). Based on the same published analyses, [25] we are also sufficiently powered to detect a difference between the top PRS decile (AMD prevalence of 22.7%) and the bottom PRS decile (AMD prevalence of 0.7%). While these analyses were used for the purpose of a power calculation, we acknowledge that the study population may be younger.

### Statistical analyses

For all cases, family history of glaucoma and AMD, gender, and ancestry will be self-reported. Genetic ancestry and biological sex will also be determined from genotyping array data. Statistical analyses will be performed in *R* (RCore Team, Austria). Missing information will be treated as missing data in analyses. For association analysis, logistic or linear regression will be used, including age, genetic sex and genetic ancestry as covariates. Other confounding variables will be added when clinically and statistically appropriate. Appropriate regressions will be performed to investigate the rate of each glaucoma or AMD classification across the risk spectrum of the PRS, and to identify any additional factors which were associated with these outcomes. An individual will be defined as a glaucoma or AMD case regardless of whether one or both eyes meet diagnostic criteria.

### Study outcomes

The primary outcome will be assessing the prevalence of glaucoma and AMD between the bottom decile, middle 80% and top decile of both respective PRS spectra. We will assess the clinical sensitivity and specificity, as well as the positive and negative predictive values of each of the glaucoma and AMD PRS. Secondary outcomes will compare glaucoma suspect cases to their PRS results, compare disease prevalence with the presence or absence of various comorbid conditions, treatment intensity requirements including the number of cases with actionable disease, the rate of diagnosed versus undiagnosed disease, and the prevalence of family history. Additionally, glaucoma and AMD cases may be graded by severity, and compared to their PRS results.

## Discussion

Glaucoma and AMD are the most common causes of irreversible blindness worldwide [2]. Both conditions are highly heritable, with recognised Mendelian and complex inheritance [35, 36, 37]. There is a paucity of screening protocols for both diseases and current guidelines are not cost-effective, in part due to poor sensitivity or specificity. To our knowledge this is the first prospective study to apply PRS testing for glaucoma and AMD in individuals from the general population, specifically recruited for this purpose.

The current NHMRC screening guidelines in Australia lack specific guidance, and are mainly relevant to those with a family history of glaucoma [16]. PRS testing for glaucoma is likely to be useful for those who do not have a known family history and have an unrecognised underlying risk. These individuals are less likely to be identified early by current screening guidelines given screening at an earlier age is only recommended for those with a family history and people of African ancestry [16]. There are no current screening guidelines for AMD in Australia. Detection is reliant on an individual experiencing symptoms and seeking ophthalmic review, or opportunistic recognition of disease during a routine assessment. The findings from this study will assist in the development of better screening guidelines for glaucoma and AMD.

Currently, risk estimation for developing glaucoma and AMD is based on a combination of demographic and clinical factors. The predictive ability of polygenic risk models for POAG and AMD are well established, particularly in European populations, and are summarised elsewhere [38]. For glaucoma, risk factors include increasing age, family history of glaucoma, African ancestry, and elevated IOP [6, 7]. Genetic risk has been largely estimated through family history alone. A positive family history carried a 9-fold risk for first-degree relatives compared to controls in one study, but this required full examination of all first degree relatives rather than self-report [39]. The accuracy of self-reported family history for glaucoma has been studied and found to be an unreliable measure as many patients are unaware of family members with diagnosed glaucoma, or have erroneous views as to what caused vision loss in relatives [40]. More recent data indicates that PRS provides a more accurate representation of risk with family history in an Australian population based study [20]. Several risk calculators have been developed to aid clinicians in screening and treatment decisions, however there remains no consensus regarding optimal timing and frequency of population screening for glaucoma [41, 42]. PRS provides a more accurate estimation of risk than traditional methods alone, with risk prediction optimised when all factors are combined [20]. AMD risk involves an interplay of genetic and environmental factors. There are several recognised environmental risk factors including age and smoking, with sex, ancestry, cardiovascular disease, and diet also suggested to be implicated [29]. A prediction model incorporating genetic, demographic and environmental risk factors was independently associated with incidence and prevalence of advanced AMD, all with strong predictive power [43]. Effective risk algorithms incorporating environmental, clinical and genetic risk factors will need to be developed. While environmental and clinical risk factors may change over time, the genetic contribution to overall risk will remain constant given genetic disease liability is fixed from conception. Therefore, an important benefit of polygenic risk testing is that PRS can be calculated at any stage of life and may be useful to inform disease prognosis and response to treatment before individuals exhibit vision loss.

Glaucoma genetic testing is currently limited to Mendelian genes (e.g. *MYOC)* which explain less than 5% of adult onset glaucoma [10, 11]. PRS testing, however, captures a much larger component of glaucoma genetic risk. Those with high polygenic risk had a comparable glaucoma risk to those with the most common Mendelian variant (OR 2.77 vs. OR 4.19), as well as being ~ 15 times more prevalent [10]. At present, genetic testing for AMD is not recommended and exists predominantly in research contexts [29, 44, 45] . Direct to consumer tests incorporating various PRS tests for both diseases are available, however these lack prospective evidence demonstrating their effectiveness [46, 47]. This study will assess the clinical validity of PRS testing in a sample representative of the general population in Australia in order to determine its application in the community.

We have previously demonstrated strong interest in polygenic risk testing for glaucoma among various groups, including those with diagnosed glaucoma, those with a first-degree relative with glaucoma, and those without any personal or family history of the condition [30, 48]. Although PRS testing for glaucoma was theoretically accepted, we identified a number of concerns and potential barriers to implementation, including residing in a rural location and unwillingness to pay for testing. There are a number of additional questions which must first be addressed before PRSs can be integrated into clinical practice.

Firstly, results must lead to actionable and cost-effective measures. Guidelines will be needed to clarify which PRS classifications warrant intervention. Those identified to be at high risk for developing glaucoma or AMD may receive more regular follow-up with an optometrist or ophthalmologist, allowing for timely treatment initiation. Treatment may be commenced before the disease becomes symptomatic. Early interventions for glaucoma may include topical IOP-lowering medication or laser therapy. Earlier surgical intervention may be considered for those with a PRS indicating a likelihood to progress rapidly or to advanced disease. While treatment options for early AMD are lacking, there are a large number of treatments under research including various pharmaceutical agents, gene therapies and surgical interventions [49]. Antioxidant supplements based on the Age-Related Eye Disease Studies (AREDS) may have benefit in those with intermediate disease in one or both eyes to reduce the risk of progressing to late AMD, or in those with late stage disease in only one eye to reduce the risk of developing it in the other eye [50]. Smoking is the only established modifiable risk factor for AMD, with the risk of progression to neovascular AMD shown to be double for those who had ever smoked [51]. Despite there being few treatment options for AMD, risk factor modification and antioxidant supplementation may still be valuable interventions in high-risk individuals. Progression from early to advanced AMD may occur rapidly and result in severe vision loss if treatment is delayed. Using tools such as an Amsler grid, individuals who are recognised to be at higher risk of this occurring could be educated to self-monitor for progression, with a pathway to access rapid assessment if symptomatic. Conversely, PRS may prevent unnecessary follow-up or treatment in those presumed to be at higher risk based on traditional risk prediction models. This may improve the cost-effectiveness of the PRS.

Secondly, it will be critical to develop frameworks which allow PRS results to be reported and communicated in a meaningful manner. Pilot reports need to be developed and tested to assess communication preferences and understanding of reported results among different stakeholders, including patients and healthcare professionals. We have previously demonstrated that the preferred method of receiving results may depend on the result itself, so report content and structure will likely vary depending on risk classification [30, 48]. This study will form the foundations of future research to develop our understanding of the clinical implementation of PRS testing for glaucoma and AMD.

Finally, there are a number of health economic elements which need to be considered before implementing PRS into clinical practice. Population-based screening for glaucoma or AMD is not currently cost-effective, so public health frameworks need to be developed which allow identification of those at increased risk while also ensuring adequate access to further treatment. Disease prevention is at the forefront of public health policy, and polygenic risk stratification has the potential to enhance primary, secondary and tertiary facets of this. Ultimately, enhanced disease screening will minimise the personal and economic costs of significant vision loss. Improved risk stratification will alleviate workload created by over investigation and treatment of those at high risk calculated using traditional risk factors, but at low genetic risk. However, it will be important to integrate genetic risk with clinical or environmental risk factors. Individuals with a strong family history would still be recommended to have regular clinical testing, even if shown to have a low PRS, due to the influence of Mendelian variants or other factors not covered by the PRS. We have shown that financial implications appear to be important to people and while some are unwilling to pay for testing the majority of individuals would be prepared to pay varying amounts [30]. Subsidisation may improve uptake, however will only be an option if it is cost-effective for the healthcare system which remains to be demonstrated.

Current PRSs for glaucoma or AMD are based on predominantly European populations and have not yet been comprehensively tested across other ethnicities. Individuals of non-European ancestry are not excluded from the study, although the accuracy of their risk predictions may be reduced. Better validation of a single pan-ancestry PRS, or ancestry-specific scores covering all ethnicities, are a major unmet need to avoid future health disparities.

In conclusion, this prospective study aims to demonstrate the clinical validity of PRS to stratify individuals from the general population and identify those who are at high risk of developing glaucoma or AMD. This will help to move towards the implementation of PRS into clinical practice and provide an objective screening tool for glaucoma and AMD. The ability to identify at-risk individuals will allow for closer monitoring and timely intervention, and ultimately reduce irreversible vision loss. Further studies will need to look into how PRS testing could alleviate some of the socioeconomic burden resulting from vision loss. The outcomes from this study will form the basis for future interventional studies to further enable a shift in the detection, treatment and prevention of diseases with complex inheritance.

## Data Availability

Data sharing is not applicable to this article as no datasets were generated or analysed during the current study.

## List of abbreviations

POAG: Primary open-angle glaucoma
AMD: Age-related macular degeneration
PRS: Polygenic risk score
IOP: Intra-ocular pressure
VEGF: Vascular endothelial growth factor
GWAS: Genome-wide association studies
SNP: Single nucleotide polymorphism
MTAG: Multitrait analysis of GWAS
OCT: Ocular coherence tomography
OAG: Open-angle glaucoma
